# Toward Minimally Invasive Extracorporeal Circulation in Oncologic Cardiac Surgery

**DOI:** 10.21470/1678-9741-2020-0389

**Published:** 2021

**Authors:** Ignazio Condello, Giuseppe Santarpino, Marco Moscarelli, Giuseppe Nasso, Giuseppe Speziale

**Affiliations:** 1 Department of Cardiac Surgery, Anthea Hospital, GVM Care & Research, Bari, Italy. E-mail: ignicondello@hotmail.it; 2 Paracelsus Medical University, Nuremberg, Germany.; 3 Department of Experimental and Clinical Medicine, Cardiac Surgery Unit, "Magna Graecia" University of Catanzaro, Italy.

Cancer and heart disease often coexist. For patients requiring open-heart surgery, this raises concern that the use of cardiopulmonary bypass (CPB) may cause a transient immunosuppression with potential to promote the spread and growth of coexisting cancer cells^[1]^.

Several series indicated that surgical stress suppresses the immune system and that the use of CPB, cardiac arrest, and ischemia contributes to this. The most significant contributors to immune suppression are still yet to be determined. It seems that the survival of cancer patients who undergo cardiac surgery is more closely related to the progression of the tumor than the surgical procedure^[2]^. Further research is needed to know whether the transient immunosuppression associated with CPB can promote the spread and growth of pre-existing cancer cells, emphasizing that lung cancer and skin melanoma have had the most extensive association studies. In conclusion, adverse effects of CPB on cancer prognosis are expected, but have not been confirmed. We read with great interest the article: "Cardiopulmonary Bypass and Cancer Dissemination: A Logical But Unlikely Association" by Braile DM and Évora PRB^[2]^. In this context, we present a letter to the editor on the potential role of minimally invasive extracorporeal circulation technologies (MiECT) in oncologic cardiac surgery. MiECT include a number of interventions aimed to reduce the clinical impact of CPB and to consequently contain postoperative morbidity and mortality. In the past, different kinds of CPB circuits were defined as "minimally invasive CPB", creating a confused scenario. Recently, the definition of a minimally invasive extracorporeal circulation (MiECC) system was standardized.

A MiECC system must include: (I) a closed CPB circuit; (II) biologically inert blood contact surfaces; (III) reduced priming volume; (IV) a centrifugal pump; (V) a membrane oxygenator; (VI) a heat exchanger; (VII) a cardioplegia system; (VIII) a venous bubble trap/venous air removing device; and (IX) a shed blood management system. Thus, this multidisciplinary perioperative strategy for attaining "more physiologic" cardiac surgery literally advances MiECC from a circuit to therapy: the circuit is the base, the peripherals (autotransfusion device, in-line monitoring sets, anaesthetic protocols, transoesophageal echocardiography, etc.) upgrade this to a system, while a multidisciplinary strategy encompasses all and renders MiECC use a holistic approach to cardiac surgery. Such strategy encounters the surgeon's and anaesthesiologist's particular techniques, with implementation of goal-directed perfusion from the perfusionist's perspective and point-of-care heparin/protamine and coagulation management from the anaesthesiologist's perspective. The ultimate goal of such policy when using MiECC is to operate on oncologic and fragile patients and to perform complex procedures as comfortable, in terms of haemodynamic and metabolic integrity, as operating on a low-risk case. This is because a "more physiologic" intraoperative perfusion is of paramount importance in this particular setting for optimizing outcome^[3]^. Reviewing the literature, we believe that in patients with cancer disease candidates for life-saving cardiac surgery procedures, MiECC technique could offer new perspectives in survival compared to conventional CPB. Further studies are needed to strengthen this perspective, between minimally invasive approach and immune preservation.
